# The complete mitochondrial genome of *Pseudocrangonyx daejeonensis* (Crustacea: Amphipoda: Pseudocrangonyctidae)

**DOI:** 10.1080/23802359.2018.1495116

**Published:** 2018-07-31

**Authors:** Chi-Woo Lee, Takafumi Nakano, Ko Tomikawa, Gi-Sik Min

**Affiliations:** a Department of Biological Sciences, Inha University, Incheon, Korea;; b Department of Zoology, Graduate School of Science, Kyoto University, Kyoto, Japan;; c Department of Science Education, Graduate School of Education, Hiroshima University, Higashihiroshima, Japan

**Keywords:** Complete mitogenome, Amphipoda, *Pseudocrangonyx daejeonensis*, stygobitic

## Abstract

The complete mitogenome sequence of a subterranean pseudocrangonyctid amphipod, *Pseudocrangonyx daejeonensis*, was determined. The complete mitogenome of *P. daejeonensis* was 15,069 bp in length with the typical 13 protein-coding genes (PCGs), 22 transfer RNAs (tRNAs), 2 ribosomal RNAs (rRNAs), and a control region (CR). This is the first complete mitogenome sequence in the family Pseudocrangonyctidae. Interestingly, gene arrangements of most amphipod species were almost identical to the typical pan-crustacean ground pattern, whereas two PCGs, both of rRNAs and CR were translocated in *P. daejeonensis*. A maximum-likelihood tree, constructed based on 30 eumalacostracan mitogenomes, confirmed that *P. daejeonensis* is closely related to the crangonyctid *Stygobromus indentatus* and *S. tenuis potomacus* and supported the monophyly of the superfamily Crangonyctoidea.

The amphipod superfamily Crangonyctoidea consists of 14 families, and its members are usually found in freshwater epigean habitats (Väinölä et al. 2008; Lowry and Myers 2012). The species belonging to the several crangonyctoidean families, e.g. Pseudocrangonyctidae and Crangonyctidae, inhabit groundwater habitats in caves, springs, and hyporhea (Holsinger 1994). Although the previous study showed the monophyly of Crangonyctidae + (Pseudocrangonyctidae + the Icelandic subterranean *Crymostygius*) (Sidorov and Gontcharov 2015), the precise phylogenetic relationship of the 14 crangonyctoidean families remains unresolved.

The genus *Pseudocrangonyx* now contains 25 species (Tomikawa and Nakano 2018), and its members are mainly distributed in subterranean waters and springs in East Asia, i.e. Eastern China, Korean Peninsula, Japan, and Russian Far East (Sidorov and Holsinger 2007; Tomikawa et al. 2016; Zhao and Hou 2017). Previous studies focusing on stygobitic amphipod species distributed in Europe and North America shed light onto the variability of the mitogenome gene order in Amphipoda (Bauzà-Ribot et al. 2009; Aunins et al. 2016). Accordingly, it is feasible that mitogenome information of the Asian *Pseudocrangonyx* species will lead us a further understanding of the evolutionary history of the gene arrangement of this peracaridan group.

Individuals of *Pseudocrangonyx* were collected from interstitial water in Daejeon, Korea (36°15.65′N, 127°20.43′E). Mitochondrial DNA extraction, sequencing, and gene annotation were performed using the methods described by Song et al. (2016). The extracted mitochondrial DNA has been kept in the DNA collection at the National Institute of Biological Resources, Incheon, South Korea (deposit no. NIBRGR0000426835). A maximum-likelihood tree was constructed using IQ-tree 1.6.3 with mtZOA + F+R6 model (Nguyen et al. 2015; Chernomor et al. 2016) based on the concatenated sequences of 10 PCGs (*atp6, cox1, cox2, cox3, cytb, nad1, nad2, nad3, nad4,* and *nad5*) from 30 eumalacostracan species including the present sequence and three isopods, and two mysids as an outgroup taxa. The *cox1* sequence of the extracted DNA was concordant with those from the type series of *Pseudocrangonyx daejeonensis* (INSDC accession nos. LC322137, LC322141; Lee et al. 2018), and thus the taxonomic identity of the present material was unquestionably clarified.

The complete mitogenome of *P. daejeonensis* (INSDC accession no. MH229998) was 15,069 bp in length and contained the typical 13 PCGs, 22 tRNAs, 2 rRNAs, and a CR. In the gene arrangement of *P. daejeonensis*, two PCGs (*nad5* and *nad1*), two rRNAs and a CR were translocated when compared with the typical pan-crustacean ground pattern. Since this is the first complete mitogenome assessed among the pseudocrangonyctid species, it should be necessary to clarify whether the gene order determined in this study is unique in *P. daejeonensis*, or representative feature of subterranean pseudocrangonyctid species.

The obtained maximum-likelihood tree showed that *P. daejeonensis* formed a clade with the crangonyctid *Stygobromus indentatus* and *S. tenuis potomacus*; this relationship recovered the monophyly of Crangonyctoidea under Gammarida defined by Lowry and Myers (2013) (Figure 1).

**Figure 1.**
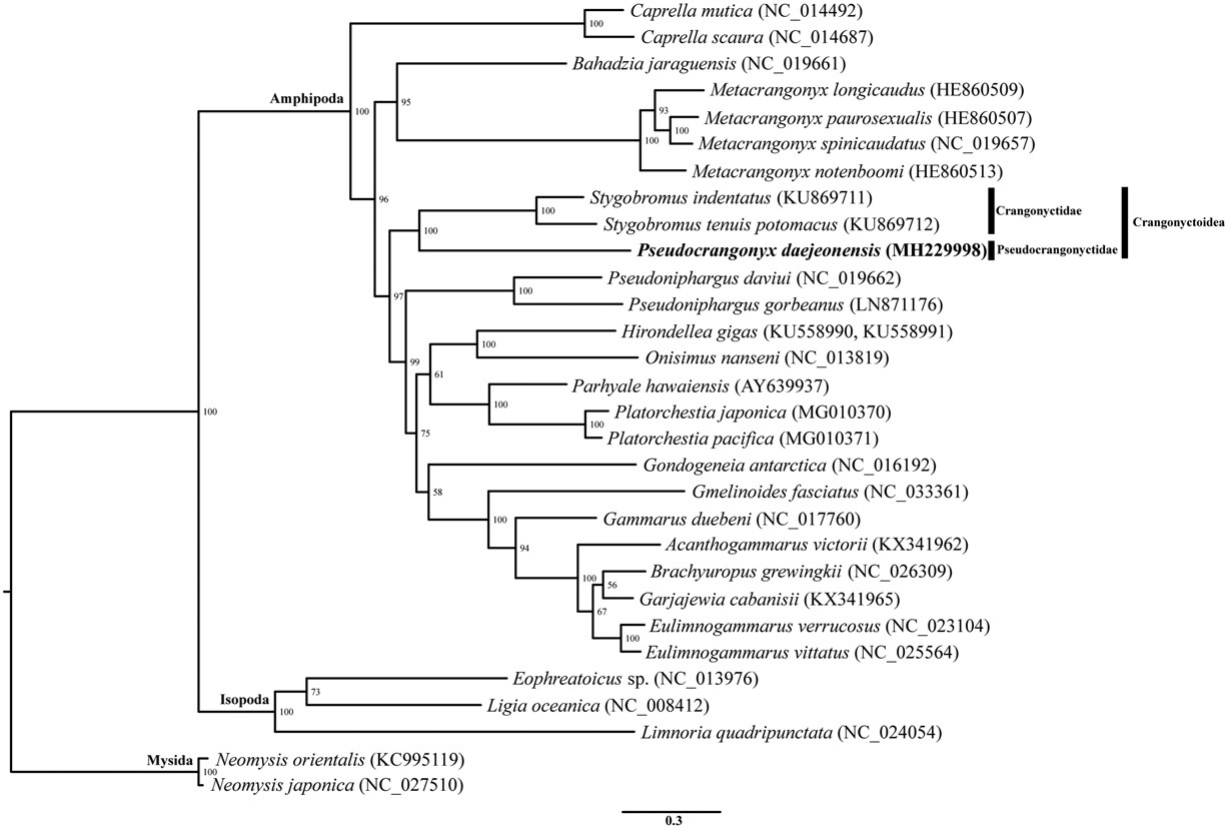
Maximum‐likelihood (ML) tree based on the mitogenome sequence of *Pseudocrangonyx daejeonensis* (MH229998) with 29 other eumalacostracan species. The bootstrap supports are shown on each node.
